# Gene susceptibility identification in a longitudinal study confirms new loci in the development of chronic obstructive pulmonary disease and influences lung function decline

**DOI:** 10.1186/s12931-015-0209-3

**Published:** 2015-04-18

**Authors:** Jungang Xie, Hongxu Wu, Yuzhu Xu, Xiaojie Wu, Xue Liu, Jin Shang, Jianping Zhao, Junling Zhao, Jianmiao Wang, Charles S Dela Cruz, Weining Xiong, Yongjian Xu

**Affiliations:** Department of Respiratory and Critical Care Medicine, Tongji hospital, Tongji Medical College, Huazhong University of Science and Technology, Wuhan, China; Section of Pulmonary and Critical Care Medicine, Department of Internal Medicine, Yale University School of Medicine, New Haven, USA

**Keywords:** Chronic obstructive pulmonary disease, Cross-sectional, Longitudinal, Gene susceptibility, Lung function

## Abstract

**Background:**

To identify COPD associated gene susceptibility and lung function in a longitudinal cohort including COPD and subjects who were at risk for developing COPD, and to replicate this in two cross-sectional and longitudinal populations in Chinese Han population.

**Methods:**

Three cohorts were recruited in this study, including an 18-year follow-up population (306 COPD and 743 control subjects) in one village in 1992 and it changed to 409 COPD and 611 controls in 2010, a 2 year follow-up study in another village (374 COPD and 377 controls) and another 2 year follow-up one in a city (541 COPD and 560 controls) in 2010. Sixteen candidate single nucleotide polymorphisms (SNPs) were selected for genotyping. Among them, 5SNPs in or near HHIP, 1SNP in IREB2 and 1SNP in FAM13A were previously reported to be associated with COPD susceptibility or lung function decline. And another 9SNPs were selected from HapMap website as HHIP tags. In 2010, totaling 1,324 COPD patients and 1,548 healthy controls were finally included in our genetic susceptibility analyses.

**Results:**

We identified two new regions showing an association with COPD susceptibility in the Human Hedgehog interacting protein (HHIP) rs11100865 and rs7654947, and we confirmed that the family with sequence similarity 13 member A gene (FAM13A) rs7671167 was associated with the development of COPD in Chinese Han population. And the HHIP rs7654947 and FAM13A rs7671167 were associated with lung function decline, and this result was replicated in other two populations.

**Conclusions:**

These results suggest an important role of the HHIP and FAM13A regions as genetic risk factors for COPD development and lung function decline in Chinese Han population. Future research on these genes should focus on the molecular mechanisms of these genes on developing COPD and creating therapies to alleviate reduced lung function.

**Electronic supplementary material:**

The online version of this article (doi:10.1186/s12931-015-0209-3) contains supplementary material, which is available to authorized users.

## Background

Chronic obstructive pulmonary disease (COPD) is a leading global cause of morbidity and mortality, and will become the fourth leading cause of death by the year 2030 [1]. Although cigarette smoking is the main pathological driver of COPD, and more than 80% COPD patients are smokers, only a small proportion of smokers develop clinically significant COPD. This suggests that a genetic predisposition could explain why only a proportion of cigarette smokers develop COPD. The alpha 1-antitrypsin gene is the only gene that has been definitely proven to influence COPD susceptibility, and present in only 1-3% of individuals with COPD [2].

Recently, some large-scale genome-wide association studies (GWAS) on COPD have identified susceptible genes and loci associated with COPD [3-9], and these genes play an important role in regulating morphogenesis and lung development, and are associated with the presence of COPD and lung function decline. The variants of these genes may influence lung development and are possibly related to COPD phenotype. However, all the current gene susceptibility studies [3,4,7,10] in COPD are cross-sectional, and smokers without COPD were chosen as the controls. In the study period, these “normal” controls without COPD may develop COPD after 10 or 20 years. Therefore, there is likely to be some bias with the cross-sectional studies in that these control subjects may not necessarily lack the susceptibility genes.

Moreover, GWAS on cross-sectional or longitudinal lung function have identified some genes which are associated with lung function, several previously unrecognized loci related to two clinically important pulmonary functions in a cross-sectional analysis [6,11-13]. Since these studies only reflected the association of these genes and lung function in the study period, they are limited for evaluating the value of the screened genes involved in the decline of lung function. Novel genes have also been identified by GWAS on longitudinal lung function in COPD patient. However, the population which was recruited in these studies either did not have any disease manifestations, or other respiratory diseases such as asthma, or they did not have any subjects who had a high risk of developing COPD in their studies of lung function follow-up [14-16]. Thus, genes identified in these studies may not accurately reflect the association with lung function decline in COPD. Our study focuses on a fixed cohort in a Chinese village that includes subjects with COPD and control subjects with similar risk factor for developing COPD. These individuals were followed for up to 18 years. We also replicated the study in two other validation cohorts with extensive follow-up information and determined the association of the genes with forced expiratory volume in one second (FEV_1_) decline.

Our research presented here highlights an approach that takes advantage of genome-wide association analyses using longitudinal and extensive follow-up data to identify susceptibility genes in COPD that influence lung function decline. This study identified two new loci and confirmed one locus with the development of COPD, and revealed one previously unreported locus and a third locus associated with lung function in three independent follow-up study cohorts.

## Materials and methods

### Study design and recruitment of subjects

**Figure 1:**
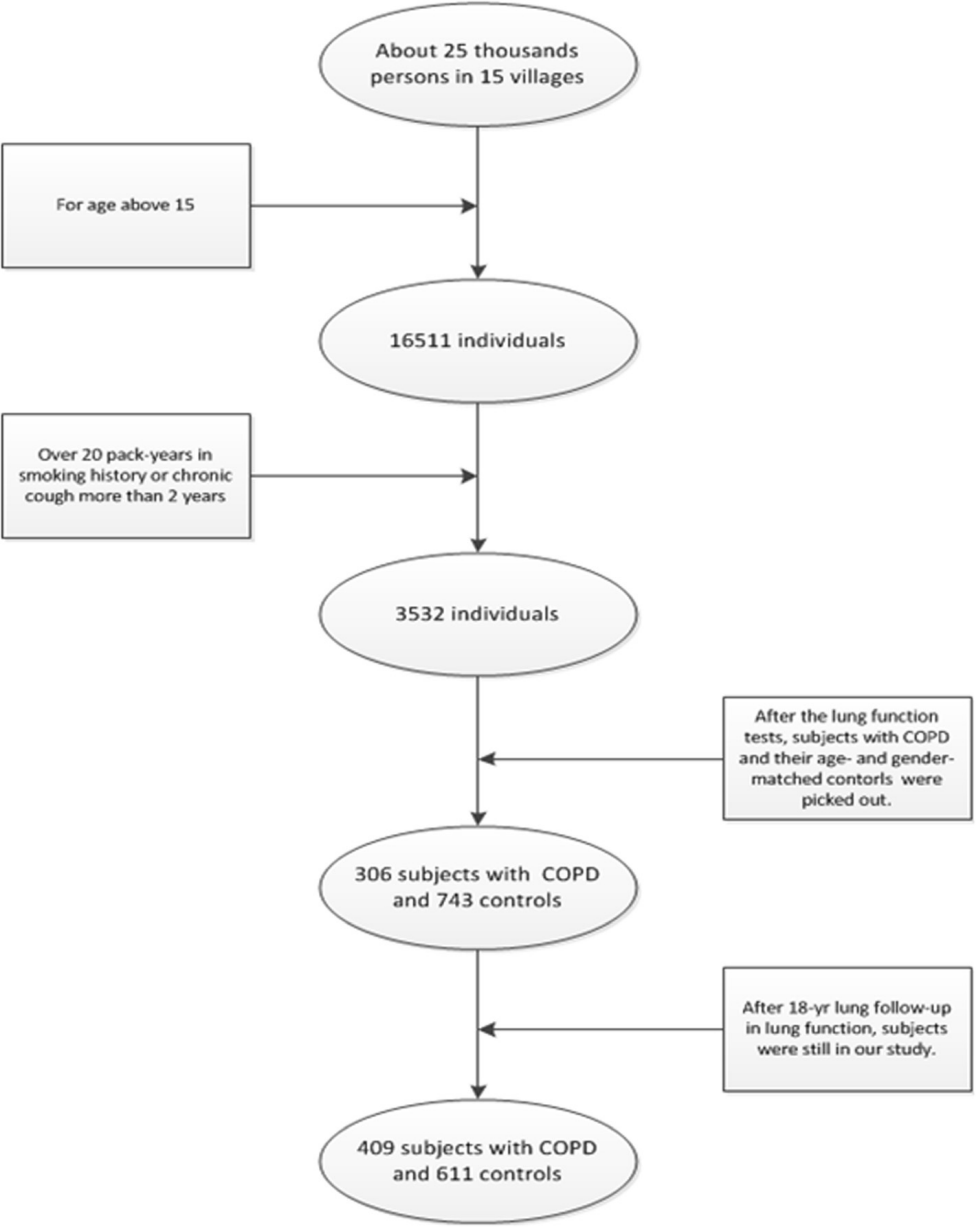
The process of our study in the prospective follow-up stage. In the beginning (in 1992), COPD and controls who had same risk for developing COPD were identified after the lung function performed. In the 18-year follow-up, some controls developed into COPD.

In the first stage, study subjects were recruited from 15 villages in Haokou, Qianjiang, Hubei, China. We screened subjects, from the population of 15 villages totaling 25,000 people to determine the risk groups and subjects with COPD, as described in Figure 1. Of the 16,511 adults, 3,532 had a chronic cough longer than 2 years or a smoking history above 20 pack-years, who might be suffering from COPD already or were at risk. After performing lung function tests, as recommended by the American Thoracic Society /European Respiratory Society [17], 306 subjects with COPD and 743 age- and gender- matched healthy subjects were enrolled in the study after their consent was provided. The inclusion and exclusion criteria of COPD subjects in this study were described previously [18]. This was an 18-year longitudinal prospective study on COPD gene susceptibility of the Chinese Han population which all subjects had a lung function follow-up observation, and were tested every five years. In the 18 year follow-up observation period, there were cases and control subjects who could not be enlisted through to the end. The main reasons for the losses were that 9 cases and 16 control subjects suffered from cancer or died with unknown causes. A few individuals withdrew their consent or moved away which resulted in lost contacts. Ultimately, there were 1,020 subjects who were enrolled and genotyped for the study.

The replication cohorts were recruited from two different environmental living conditions and locations that were different from the first cohort. The subjects with COPD in the rural replication cohort were enlisted from scattered rural areas in Hubei province except Haokou. In the two replication cohorts, a total of 374 cases and 377 controls from the rural cohort, and 541 cases and 560 controls from the urban cohort in 2010 were enrolled and 205 COPD patients underwent lung function follow-up observations for approximately 2 years. The association of FEV_1_ decline with genetic variants was also replicated in these individuals.

All the research procedures performed in this study were screened and approved by the Research Ethics Committee, Huazhong University of Science and Technology, Wuhan, China.

### SNPs selection and linkage disequilibrium analysis

**Figure 2:**
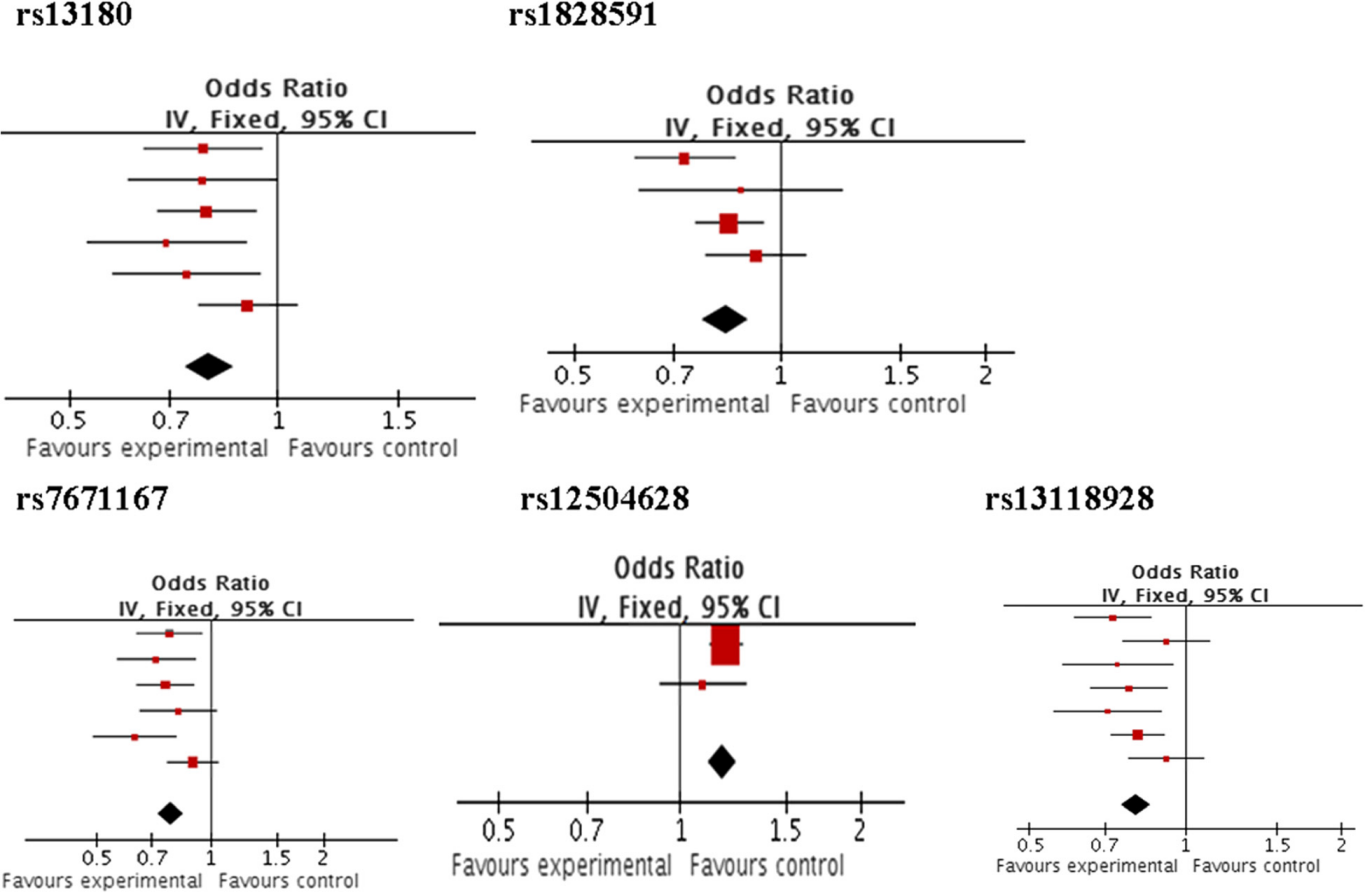
The meta-analysis on the associations of SNPs with lung function or COPD susceptibility.

There were 5 single nucleotide polymorphisms (SNPs) in or near HHIP (rs1980057, rs12504628, rs13147758, rs13118928, rs1828591), 1SNP in IREB2 (rs13180) and 1SNP in FAM13A (rs7671167) genotyped in our study. These have been reported to be associated with COPD susceptibility or lung function in Caucasians and in the meta-analysis studies were shown as probable risk genetic variants related to COPD or lung function decline. In meta-analysis, the SNPs in HHIP, FAM13A and IREB2, which were reported in different study cohorts that focused on lung function or COPD susceptibility [10,19-22] were analyzed for their odds ratios (ORs) as shown in Figure 2 (full details of meta-analyses can be accessed in Additional file 1). Some of the cohorts analyses were performed in Chinese Han population in these reports, however, the number of subjects was not large enough [23,24]. Moreover, 60SNP markers with a minor allele frequency (MAF) ≥0.05 in or near HHIP were downloaded from HapMap (http://www.hapmap.org/) for the Chinese Han population (Chinese Han from Beijing–CHB). Tag SNPs were selected for the gene by using Tagger in Haploview (http://www.broadinstitute.org/haploview/haploview). Under the pair-wise mode used, a minimal set of markers were selected. All the captured alleles were correlated at an r^2^ ≥ 0.8 with a marker. Nine tagged SNPs (rs7654947, rs11100865, rs6819412, rs7689420, rs7675744, rs6826012, rs2639576, rs6812389, rs1489758) were selected in the HHIP region in CHB. Taken together, a total of 14SNPs in or near the HHIP, 1SNP in IREB2 and 1SNP in FAM13A were selected for genotyping in our study.

The linkage disequilibrium (LD) between the SNPs in the HHIP locus were examined with the Haploview 4.2 program (Broad Institute of MIT and Harvard, Boston, MA, USA) for all control subjects [25].

### Genotyping and quality control

Genomic DNA was extracted from peripheral venous blood, which was drawn from each subject, after their informed consent was obtained, by using the Blood Genomic DNA Purification Kit (Tiangen Biotech, Beijing, China) according to the manufacturer’s protocol. 16 single nucleotide polymorphisms (SNPs) in genes which have been reported to be associated with COPD susceptibility or decreased lung function in Caucasians [8,20,21,26] were chosen for the genotyping tests in all subjects, who were genotyped using the TaqMan Genotyping system (Applied Biosystems, Foster City, CA) without knowledge of the case or control status of the subjects. The PCR were performed on the PCR 9700 machine (Applied Biosystems, Foster City, CA) under the following conditions: 95°C for 10 min followed by 45 cycles of 92°C for 15 s and 60°C for 90s. After the reactions were completed, the DNA chips were read under Detection System of OpenArrayNT (BioTrove, Baltimore, Maryland, United States). The data were analyzed using the TaqMan Genotyper Software version 1.2 (Applied Biosystems, Foster City, CA). Subjects with a call rate of < 95%, and SNPs with a call frequency < 95% were removed.

### Statistical analysis

The differences in the distribution of categorical variables in characteristics of subjects were tested using the Pearson’s chi-square test and continuous variables by Student’s *t* test. The Hardy–Weinberg equilibrium was assessed by the goodness-of-fitness chi-square test to compare the observed genotype frequencies to the expected genotype frequencies in the controls.

Logistic regression was used to evaluate the associations between SNPs of HHIP, IREB2 and FAM13A genotypes and COPD susceptibility. The odds ratios (ORs) and their 95% confidence intervals (CIs) were calculated after adjustments for age, sex and smoking history. The comparisons of FEV_1_ decline in individuals with different genotypes of SNPs were inspected by student’s *t* test when the data met the normal distribution and were expressed as means ± SEMs. All statistical analyses were conducted by using Statistical Package for the Social Sciences (SPSS) version 19.0 software (SPSS Inc., Chicago, IL, USA) or GraphPad Prism 5 software (GraphPad, San Diego, CA).

## Results

### Characteristics of subjects in the study

Table 1 presents the characteristics of individuals participating in the study that included the 18-year follow-up observations and the two replication cohorts. In the subjects who completed the study, a total of 306 cases (286 males) and 743 controls (684 males) with a mean age of 46.07 ± 8.68 years in 1992 were enrolled in the first stage. A total of 112 healthy subjects in 1992 were found to have obstructive pulmonary ventilation disorders after lung function tests in 2010 and this group of subjects we designated as cases to compare with our controls. We had 409 (385 males) cases and 611 controls (566 males) with age of 63.70 ± 8.86 years who remained in the study in 2010. We selected age- and gender- matched controls for the cases in each group to perform the genotype tests from the corresponding healthy group. The smoking status and history of subjects in 1992 and 2010 were nearly analogous between cases and controls and few were non-smokers.

**Table 1:** **Characterististics of groups of study subjects**

**Variable**	**Subjects in 1992**		**Subjects in2010**		**Subjects of Cases form controls**		**Rural replication subjects**		**Urban replication subjects**	
	**Cases**	**Controls**	**Cases**	**Controls**	**Cases**	**Controls**	**Cases**	**Controls**	**Cases**	**Controls**
No. of subjects	306	743	409	611	112	587	374	377	541	560
Age (±SD) (yr)	50.81 ± 6.17	49.19 ± 6.19	67.58 ± 7.12	66.93 ± 6.20	66.70 ± 6.24	66.58 ± 6.35	62.46 ± 9.28	61.39 ± 10.13	65.73 ± 9.13	64.55 ± 9.86
Male (%)	286 (93.5%)	684(92.1%)	385(94.1%)	566(92.6%)	106(94.6%)	546(93.0%)	304(81.3%)	309(82.0%)	443(81.9%)	445(79.5%)
Pack-years (±SD)	28.74 ± 16.57	25.58 ± 15.76	40.42 ± 24.40	43.29 ± 34.75	37.75 ± 18.77	40.29 ± 32.95	26.64 ± 21.13	22.74 ± 20.62	41.22 ± 48.42	45.68 ± 20.14
Post-FEV_1_ (±SD)	2.03 ± 0.52	2.82 ± 0.57	1.58 ± 0.62	2.43 ± 0.57	1.91 ± 0.61	2.45 ± 0.53	1.54 ± 0.70	2.59 ± 0.76	1.15 ± 0.58	2.31 ± 0.69
Post-FEV_1_%pre (±SD)	63.36 ± 27.01	108.15 ± 32.9	54.14 ± 22.03	97.23 ± 27.29	60.98 ± 14.43	98.01 ± 25.19	66.11 ± 25.19	106.8 ± 19.71	46.63 ± 20.26	121.3 ± 15.79
Post-FEV_1_/FVC ratio (±SD)	60.18 ± 7.36	78.70 ± 6.19	57.28 ± 9.48	78.22 ± 5.79	61.71 ± 7.15	78.22 ± 5.79	55.04 ± 11.46	78.70 ± 6.17	50.95 ± 12.47	82.34 ± 8.19

The replication cohorts, totaling 374 cases (304 males, 62.46 ± 9.28) and 377 controls (309 males, 61.39 ± 10.13), were from the rural cohort in 2010, and 541 cases (443 males, 65.73 ± 9.13) and 560 controls (445 males, 64.55 ± 9.86) were from the urban cohort in 2010.

There were no significant differences in the distribution of age and gender between cases and controls in either group of subjects.

### Genotypes and risk of COPD

In the first cohort, the regular smokers, who were considered to be in the risk groups of COPD, had lung function tests performed according to the same operational standards every five years in Haokou. There were 306 COPD patients and 743 healthy controls at the beginning of our study in 1992, but at the end of the study in 2010, there were 409 cases and 611 controls. Among the cases in 2010, there were 112 individuals who had normal lung function and were part of the healthy control group in 1992. Regardless of their status in 1992 or 2010, all subjects who had completed our study were genotyped. We compared the genotypes of cases with their gender- and age- matched healthy controls both in 1992 and 2010, especially for the cases from controls and their control subjects who still had normal lung function in 2010 (Table 2).

**Table 2:** **Characteristics of the SNPs genotype frequencies (GF) and their associations with COPD risk in different group of subjects**

**Gene**	**SNP**	**Position (NCBI)**	**Genotypes of SNPs**	**Subjects of 1992**			**Subjects of 2010**			**Subjects of Cases from Controls**		
				**GF(Cases/Contols)**	**P**	**OR(95% CI)**	**GF(Cases/ Controls)**	**P**	**OR(95% CI)**	**GF(Cases/ Controls)**	**P**	**OR(95% CI)**
HHIP	rs11100865	145870597	G → A									
			GG	0.239/0.284		reference	0.229/0.299		reference	0.213/0.282		reference
			AG	0.448/0.498	0.166	1.287(0.900-1.841)	0.477/0.492	0.262	1.266(0.838-1.912)	0.525/0.504	0.301	1.382(0.749-2.551)
			AA	0.313/0.218	0.007	1.715(1.162-2.532)	0.294/0.209	0.001	1.925(1.214-2.687)	0.263/0.214	0.17	1.637(0.809-3.313)
	rs12504628	145655774	T → C									
			TT	0.500/0.506		reference	0.519/0.497		reference	0.550/0.493		reference
			CT	0.351/0.421	0.383	0.829(0.543-1.264)	0.360/0.430	0.265	0.815(0.569-1.168)	0.375/0.430	0.477	0.829(0.493-1.392)
			CC	0.149/0.073	0.018	2.114(1.136-3.935)	0.121/0.072	0.116	1.597(0.891-2.863)	0.075/0.074	0.81	0.890(0.436-2.291)
	rs13118928	145705839	A → G									
			AA	0.470/0.457		reference	0.495/0.441		reference	0.538/0.446		reference
			AG	0.373/0.459	0.298	0.801(0.527-1.217)	0.379/0.474	0.069	0.718(0.502-1.026)	0.389/0.475	0.148	0.685(0.410-1.143)
			GG	0.158/0.083	0.048	1.839(1.005-3.365)	0.126/0.085	0.317	1.334(0.758-2.348)	0.075/0.079	0.629	0.792(0.309-2.035)
	rs7654947	145845180	T → C									
			TT	0.321/0.417		reference	0.327/0.433		reference	0.338/0.441		reference
			CT	0.440/0.402	0.112	1.433(0.920-2.235)	0.453/0.387	0.023	1.552(1.062-2.267)	0.475/0.372	0.042	1.750(1.020-3.001)
			CC	0.239/0.182	0.027	1.820(1.070-3.096)	0.220/0.180	0.004	1.882(1.192-2.816)	0.188/0.166	0.039	1.842(1.201-2.568)
FAM13A	rs7671167	90103002	T → C									
			TT	0.313/0.252		reference	0.322/0.235		reference	0.338/0.224		reference
			CT	0.463/0.442	0.43	0.832(0.527-1.314)	0.463/0.438	0.222	0.779(0.522-1.163)	0.463/0.443	0.426	0.791(0.443-1.411)
			CC	0.224/0.306	0.046	0.582(0.342-0.990)	0.215/0.327	0.002	0.480(0.303-0.762)	0.200/0.335	0.027	0.458(0.229-0.915)

As shown in Table 2, there were 4SNPs (rs11100865, rs12504628, rs13118928 and rs7654947) that occurred in or near the HHIP and one SNP in FAM13A (rs7671167) in different genotypes displaying significant differences between cases and controls in 1992. These 4SNPs occurring in HHIP were associated with increasing COPD risk after adjusting for age, gender and smoking history. One SNP in FAM13A, rs7671167, was observed as having significant associations with an increased risk of COPD.

However, it was found that genotypes of 2SNPs in or near HHIP (rs11100865 and rs7654947) and 1SNP in FAM13A (rs7671167) were associated with an increased risk of COPD in 2010. Among the 5SNPs identified in subjects of 1992, rs12504628 and rs13118928 were not significantly associated with an increased COPD risk in 2010. The SNPs in HHIP (rs11100865 and rs7654947) and in FAM13A (rs7671167) were significantly associated with risk of COPD. A relationship with increased COPD risk in the group subjects of cases from controls and their healthy controls was observed with the genotypes of these 5SNPs. The genotypes of rs7671167 revealed similar results as in subjects of 2010 displaying an association with increased COPD risk. No significant differences were found in the other 3SNPs in HHIP.

**Figure 3:**
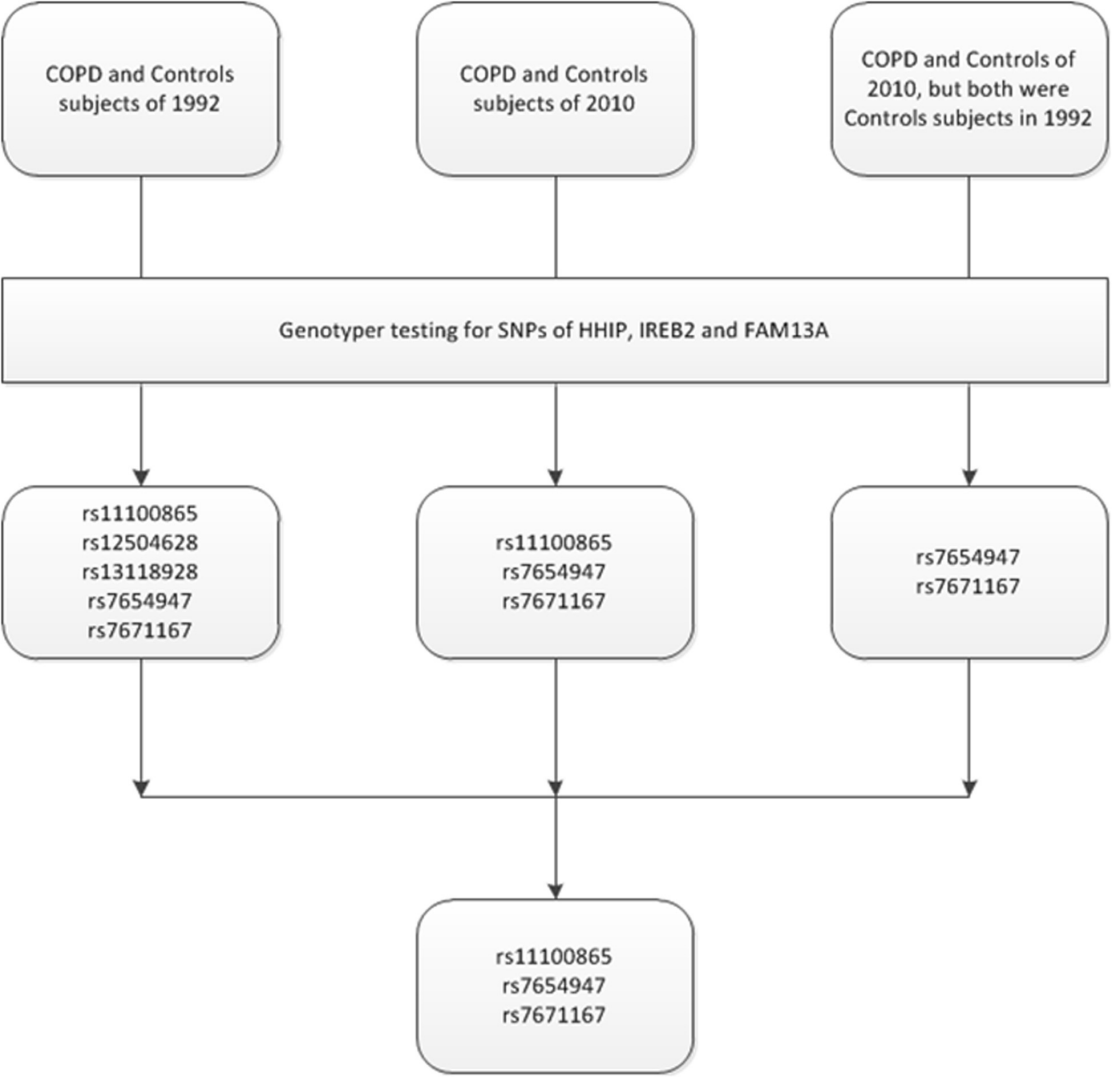
The genetic variants identified as being associated with increased COPD risk in different groups of subjects of the follow-up stage. In different periods of the first stage, the COPD risk variants were identified by genotype comparison of COPD subjects with healthy controls who were at COPD risk. By contrast, the common variants were considered to have less bias and to be more reliable. Since the cases from controls and their healthy controls in 2010 were part of the control individuals in 1992, some COPD risk variants may be not be identified.

In the 18 years of observations, some of the subjects had changed their status from healthy controls to COPD with their genetic differences playing critical roles. We analyzed the relationship of genotypes and COPD by comparing the different SNPs genotypes and COPD status, especially for subjects who, over the study period, had developed obstructive ventilatory dysfunction from normal subjects in the study. Figure 3 demonstrates the SNPs in genotypes which were associated with COPD risk were selected out in different groups, and the common genetic variants were considered to be more correlative to risk of COPD. We followed the subjects and examined their lung functions for 18 years and compared genotypes of the SNPs in each group of our study to reduce the bias that can arise from cross-sectional studies. Finally, 2SNPs in HHIP (rs11100865 and rs7654947) and 1SNP in FAM13A (rs7671167) were considered as being associated with COPD susceptibility in the Chinese Han population.

In the replication cohorts, 12SNPs were successfully genotyped. Among the five SNPs which were identified as being associated with increased COPD risk in either group of subjects in the first stage, it was shown that the genotypes in rs11100865, rs7654947 and rs7671167 were associated with risk of this disease in both the rural replication cohort and the urban cohort. This result was consistent with the cohort study in the first stage. Additionally, in urban replication cohort, the genotypes of rs13118928 were also observed as associating with an increased COPD risk.

In 2010, a total of 1,333 COPD cases and 1,552 controls in both stages of our study were added together to replicate the identified SNPs in the entire population of our study. Table 3 shows that rs11100865 and rs7654947 in HHIP and rs7671167 in FAM13A were also associated with an increased risk of COPD, and this was consistent with the discovery at the first stage which revealed the same SNPs in loci associated with COPD susceptibility. Each of these SNPs in all control subjects was in Hardy-Weinberg equilibrium.

**Table 3:** **Genotype frequencies of HHIP and FAM13A SNPs and their association with COPD risk in COPD patients and controls in replication cohorts**

**Gene**	**SNP**	**Position (NCBI)**	**Genotypes of SNPs**	**Rural replication cohort**			**Urban replication cohort**			**Subjects of all Cases and Controls**			**HWE p-value**
				**GF(Cases/Contols)**	**P**	**OR(95% CI)**	**GF(Cases/Controls)**	**P**	**OR(95% CI)**	**GF(Cases/Controls)**	**P**	**OR(95% CI)**	
HHIP	rs11100865	145870597	G → A										0.229
			GG	0.291/0.304		reference	0.297/0.380		reference	0.282/0.334		reference	
			AG	0.484/0.525	0.371	1.174(0.826-1.668)	0.475/0.421	0.031	1.375(1.029-1.838)	0.478/0.472	0.048	1.210(1.002-1.461)	
			AA	0.225/0.171	0.034	1.605(1.037-2.485)	0.228/0.199	0.079	1.373(0.964-1.957)	0.240/0.194	0.001	1.451(1.155-1.822)	
	rs12504628	145655774	T → C										0.314
			TT	0.489/0.513		reference	0.518/0.477		reference	0.509/0.493		reference	
			CT	0.439/0.383	0.318	1.174(0.857-1.608)	0.379/0.412	0.083	0.785(0.597-1.032)	0.394/0.409	0.254	0.904(0.760-1.075)	
			CC	0.073/0.104	0.225	0.709(0.407-1.235)	0.104/0.112	0.119	0.705(0.454-1.095)	0.097/0.098	0.6	0.926(0.694-1.235)	
	rs13118928	145705839	A → G										0.875
			AA	0.486/0.479		reference	0.535/0.504		reference	0.511/0.478		reference	
			AG	0.445/0.429	0.985	1.003(0.736-1.367)	0.391/0.396	0.273	0.858(0.653-1.128)	0.407/0.429	0.08	0.857(0.722-1.019)	
			GG	0.068/0.092	0.239	0.707(0.397-1.259)	0.073/0.100	0.044	0.606(0.373-0.987)	0.082/0.093	0.119	0.786(0.580-1.064)	
	rs7654947	145845180	T → C										0.370
			TT	0.369/0.514		reference	0.351/0.309		reference	0.352/0.406		reference	
			CT	0.452/0.315	1.99E-05	2.049(1.473-2.848)	0.494/0.501	0.298	0.856(0.639-1.147)	0.472/0.413	0.001	1.334(1.121-1.612)	
			CC	0.179/0.171	0.032	1.579(1.040-2.396)	0.155/0.190	0.04	0.663(0.448-0.982)	0.176/0.182	0.305	1.131(0.894-1.432)	
FAM13A	rs7671167	90103002	T → C										0.205
			TT	0.288/0.232		reference	0.323/0.266		reference	0.312/0.247		reference	
			CT	0.503/0.452	0.467	0.873(0.604-1.260)	0.503/0.495	0.488	0.899(0.666-1.214)	0.495/0.466	0.116	0.855(0.703-1.040)	
			CC	0.209/0.316	0.002	0.508(0.333-0.774)	0.174/0.239	0.029	0.661(0.455-0.959)	0.193/0.288	8.71E-08	0.531(0.421-0.670)	

Based on the 18-year follow-up and the two replication cohorts, we identified two new loci (rs11100865 and rs7654947) that were associated with increased COPD susceptibility, and confirmed that one loci (rs7671167) in FAM13A was associated with the development of COPD in Chinese Han population.

**Figure 4:**
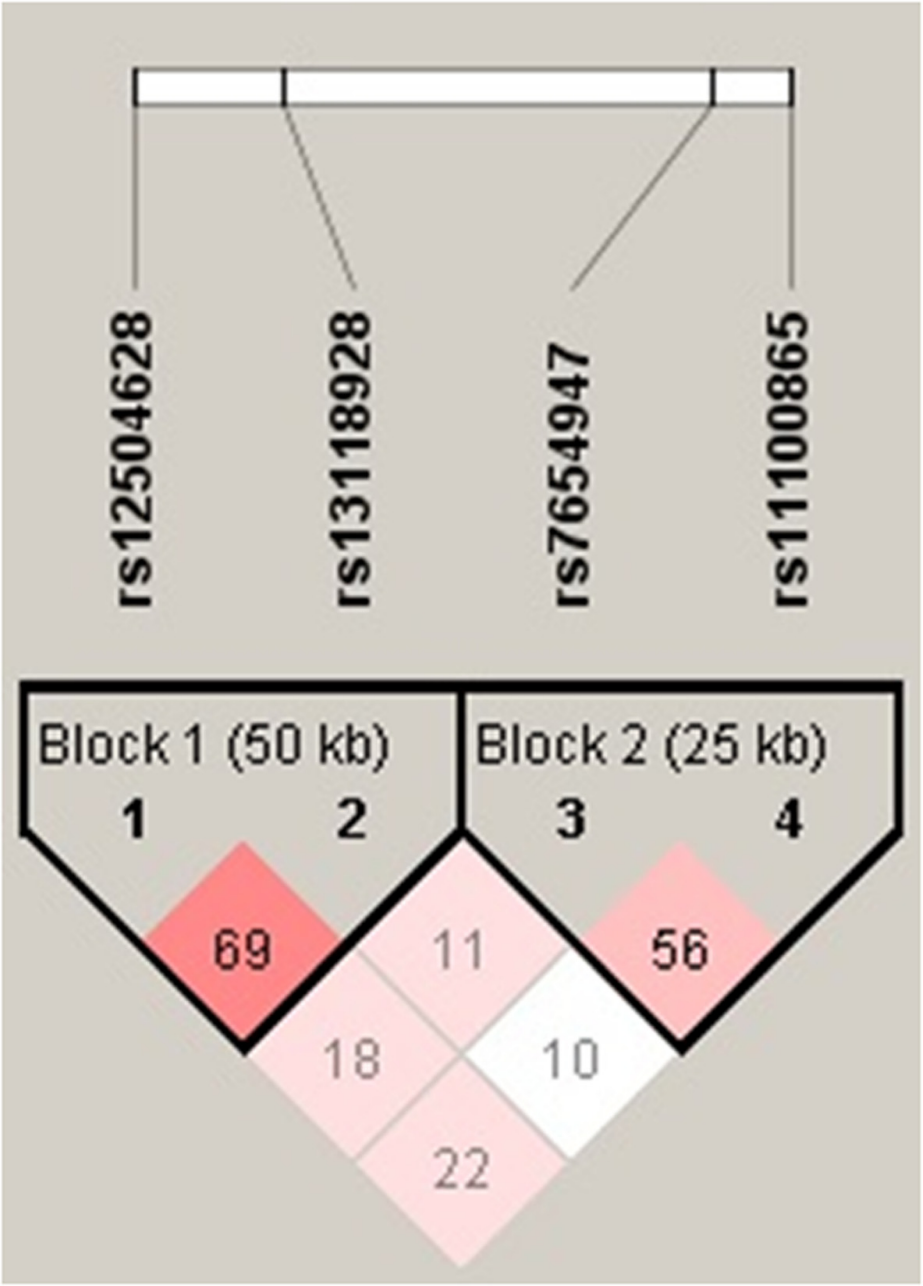
The LD values of the probable COPD risk variants in HHIP identified in all controls of our study.

The linkage disequilibrium among these SNPs in the HHIP region, which were identified as probably being associated with increased COPD susceptibility, is shown in Figure 4. The LD values were examined in all healthy controls in 2010 which includes the two replication cohorts are presented as r^2^.

### Association between decline in FEV_1_ and SNPs genotypes

**Figure 5:**
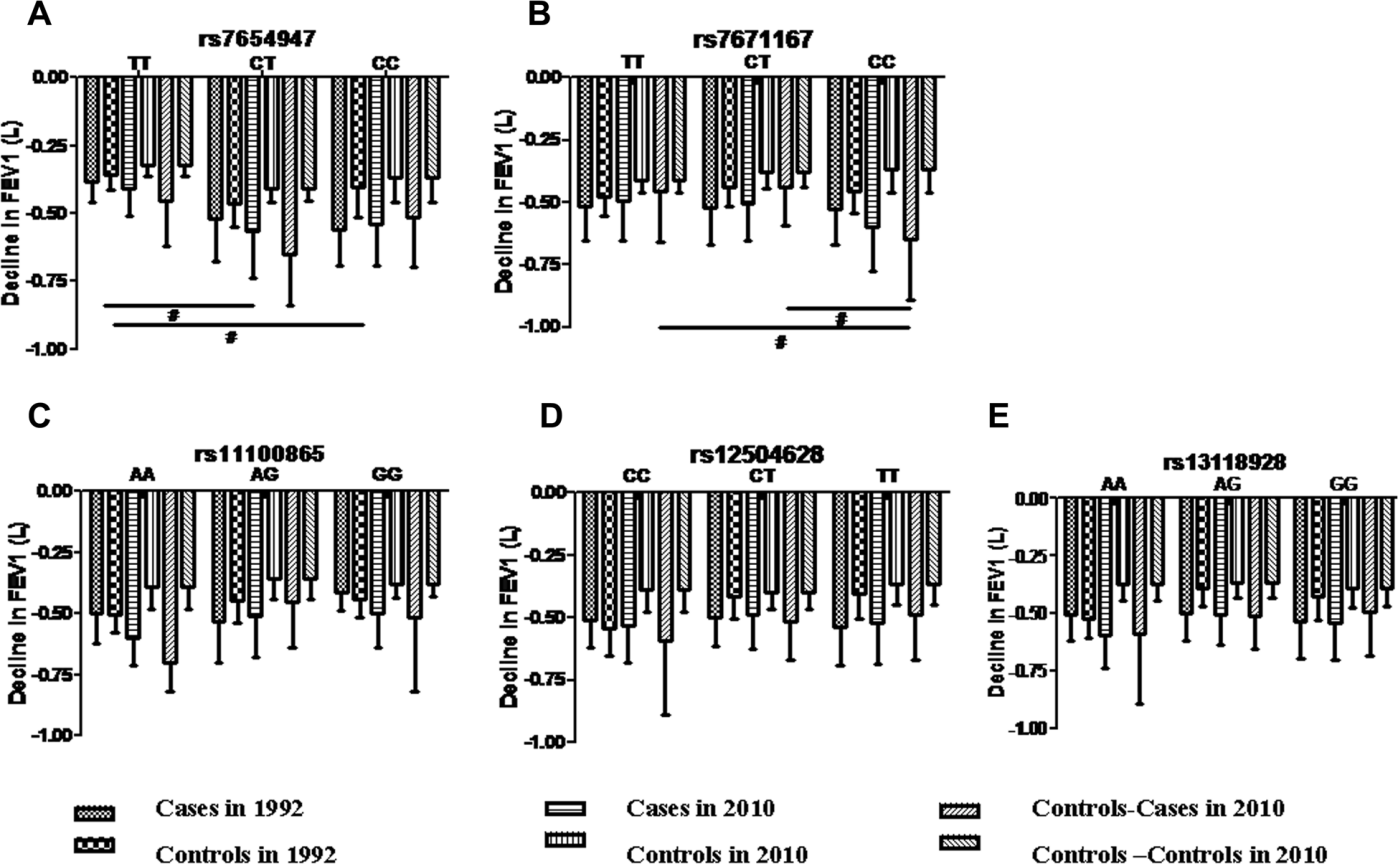
The FEV_1_ decline in different genotypes of subjects in the first stage of the 18-year follow-up. **A.** FEV_1_ decline in different genotypes of rs7654947. **B**. FEV_1_ decline in different genotypes of rs7671167. **C**. FEV_1_ decline in different genotypes of rs11000865. **D**. FEV_1_ decline in different genotypes of rs12504628. **E**. FEV_1_ decline in different genotypes of rs13118928. ^#^ p < 0.05 represents the statistical difference between individuals of different genotypes in groups of subjects.

In our research, the FEV_1_ decline in every subject over the 18 years is variable for different SNPs genotypes. The FEV_1_ decline in each genotype from the 18-year follow-up observations is shown in Figure 5. In rs7654947, the FEV_1_ decreases were higher in those CC or CT subjects than TT. There were significant differences in the groups of cases and controls in 1992 and cases in 2010 (p<0.05). In rs7671167, the FEV_1_ decreases occurred more frequently in CC subjects compared with CT or TT in cases from the controls. The difference was statistically significant (p = 0.0059). The decrease of FEV_1_ in other SNPs in different genotypes showed no significant differences in either group of subjects. The remaining 14 loci were not associated with a decline of pulmonary function (data not shown).

**Figure 6:**
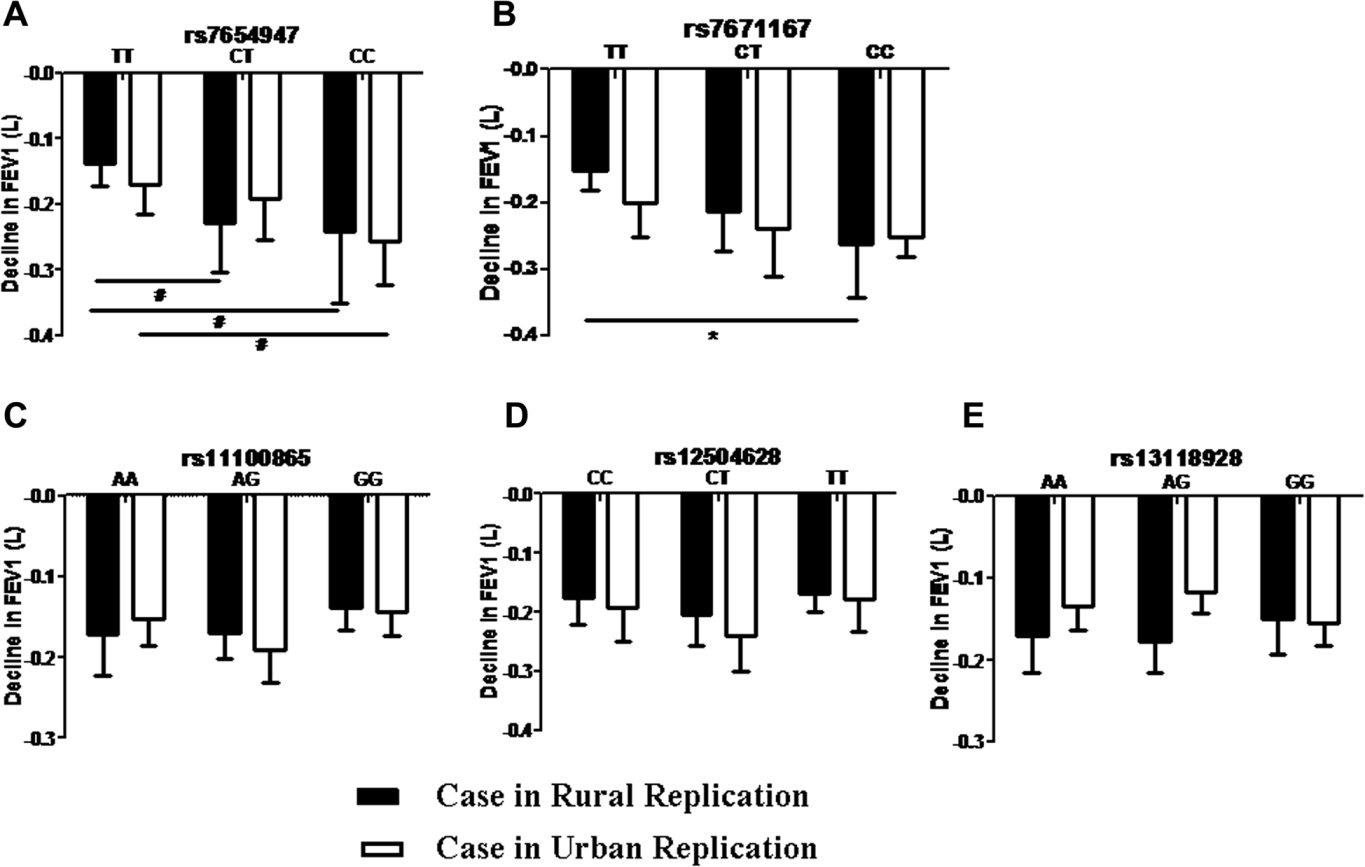
The FEV_1_ decline in different genotypes of individuals in replication cohorts in the 2-year follow-up. **A.** FEV_1_ decline in different genotypes of rs7654947 in replication cohorts. **B**. FEV_1_ decline in different genotypes of rs7671167 in replication cohorts. **C**. FEV_1_ decline in different genotypes of rs11000865 in replication cohorts. **D**. FEV_1_ decline in different genotypes of rs12504628 in replication cohorts. **E**. FEV_1_ decline in different genotypes of rs13118928 in replication cohorts. ^#^ p < 0.05, and * p<0.01, represent statistical difference in different genotypes of the follow-up replication subjects.

In Figure 6, the association of the identified COPD risk genetic variants, with a FEV_1_ decline in follow-up individuals, in replication cohorts is demonstrated. The individuals with CT and CC alleles in the rural replication cohort cases and CC in the urban replication cases of rs7654947 showed a greater FEV_1_ decline compared with the TT subjects. In rs7671167, the cases in rural replication cohort with the CC allele had a greater FEV_1_ decline compared to cases with the TT allele. In either replication cohorts (rural or urban), there was no significant differences in genotypes on the decline of FEV_1_ in other SNPs. This result was consistent with the association of FEV_1_ decline with the COPD risk variants in the first 18-year follow-up stage.

Based on our 18-year follow-up and 2-year follow-up in the two replication cohorts, we have identified one new loci (rs7654947 in HHIP) that was associated with lung function decline, and one loci (rs7671167 in FAM13A) that was associated with lung function decline and was consistent with previous research performed on Caucasians [21].

## Discussion

In our genome-wide association study on gene susceptibility of COPD comprising 306 COPD and 743 control subjects in an 18-year follow-up population, and 915 COPD patients with 937 controls in two replication cohorts of a 2-year follow-up, a total longitudinal of 1,333 COPD cases and 1,552 controls were recruited in this study. Two new loci (rs11100865 and rs7654947) in HHIP and one SNP (rs7671167) in FAM13A were identified as being associated with increased COPD risk. The SNP of rs7654947 (a new locus) in HHIP was associated with FEV_1_ decline after the 18-year follow-up observation and 2-year longitudinal observation in two replication cohorts. These differences were statistically significant.

This is the first time that a longitudinal 18 years study was applied to the gene susceptibility of COPD and lung function decline, and to demonstrate two new SNPs in HHIP that associated with COPD susceptibility. Nevertheless, the two other SNPs (rs12504628 and rs13118928) at the HHIP locus associated with risk of COPD in subjects of 1992 in the follow-up population, were previously shown to be associated with lung function and risk of developing COPD in Caucasians [19,22,27] and rs12504628 in Chinese Han population [28]. However, none of them were determined to have significant associations with lung function or COPD risk in both of the replication cohorts or the entire population. Thus, no significant difference appeared in the FEV_1_ decline of individuals in different genotypes of them, in the 18 years follow-up. Distinguishable from other GWAS performed on cross-sectional cohorts, our study is based on a prospective follow-up cohort that lasted for 18 years and has identified important genetic variants associated with increased COPD risk.

There are many common genetic variants in the loci of chromosomes 4 (HHIP and FAM13A) [21] and 15 (CHRNA3/5 and IREB2) [6,29] that display roles in the development of COPD, and the CHRNA3/5 locus that is associated with smoking addiction [10] which results in smoking-related lung diseases, such as COPD and lung cancer. Pillai et al reported that HHIP variants were associated with the systemic components and the frequency of exacerbations in individuals with COPD, and FAM13A had evidence for an association with lung function and emphysema in COPD patients [10]. Zhou et al demonstrated that SNPs near HHIP were strongly associated with pulmonary function levels and moderate-to-severe COPD in a Polish cohort [26]. A GWA study in the Framingham Heart Study reported that SNPs in or near HHIP were associated with lung function and the SNPs were different from these described above [11]. Cho et al reported that variants in FAM13A were not associated with pack-years of cigarette smoking but contributed to the development of COPD [21]. The lung function decline could also be displayed in other respiratory diseases such as asthma in addition to COPD. Both COPD and asthma share the common feature of a strong heritability for FEV_1_ with twin studies focused on lung function [30] and genetic loci were identified for COPD, asthma and lung function in GWAS. Among these, HHIP and FAM13A are associated with both COPD and lung function in individuals of European ancestry. Our study selected the SNPs frequently being identified as associating with COPD or lung function in Caucasians. Of these, we performed a meta-analysis that show them to be potentially COPD risk variants and also selected out those SNPs downloaded from HapMap for CHB in HHIP. Two SNPs (rs12504628 and rs13118928) at the HHIP locus and 1SNP (rs7671167) at FAM13A, which were chosen from those associated with an increased risk of COPD or lung function previously reported in Caucasians [19-22,27], were also associated with an increased risk of COPD in cross-sectional individuals, in 1992. However, only rs7671167 in FAM13A was successfully replicated in both the rural and urban replication cohorts. In these SNPs, which were selected from the downloaded HapMap for CHB, both of rs11100865 and rs7654947 in HHIP were identified as being associated with an increased risk of COPD in the longitudinal stage and the two replication cohorts.

Some of the healthy control subjects in 1992 developed into COPD after 18 years follow-up. After comparisons of the different groups of subjects associated with COPD susceptibility, the bias on the cross-sectional GWA study could be reduced when the relationship of genetic variants and COPD was estimated in our longitudinal stage. To determine the relationship of lung function decline and the genetic variants identified associating with COPD susceptibility in our study, we followed-up the subjects recruited in 1992 in pulmonary function observation for 18 years instead of cross-sectional lung function parameters analyzed in the GWA study. FEV_1_ is a main parameter to classify the severity of obstructive pulmonary disease and to follow its progress [31]. The FEV_1_ decreases in different genotypes of the genetic variants, which were possibly associated with COPD risk, were compared to each other to disclose the relationship of deterioration of lung function with different genotypes. Through the follow-up observation of pulmonary function decline in subjects in 18 years, the relationship of lung function and genetic variants identified in our study would be more objective and credible. Among the 4SNPs in HHIP and 1SNP in FAM13A, which were demonstrated as being related to COPD susceptibility, the genotypes of rs7654947 in HHIP and rs7671167 in FAM13A in FEV_1_ decline were significantly different in cases or controls of the follow-up cohort. Additionally, there were no significant differences detected of FEV_1_ decline in any group of cases or control subjects with genotypes of rs12504628 and rs13118928 in HHIP. Since the first GWA studies focused on lung function in the Framingham Heart Study [9], there are several large-scale research efforts involving a large number of participants in associated studies between genetic variants and lung function. The majority of these studies were cross-sectional analyses of lung function associated with susceptible genes. Nevertheless, some of these studies were carried out in follow-up cohorts to establish the novel loci influencing lung function [7,15], and the decline of lung function in participants were mainly confined in healthy individuals. The development of COPD takes time and the follow-up measures of lung function in our study lasted for 18 years, and allowed the monitoring of COPD development per 5 years for this period. Moreover, subjects in our follow-up stage were COPD patients or healthy individuals at risk of COPD, who were addicted to cigarette smoking. There is no follow-up research focused on the decline of lung function in participants who were at risk of or was already suffering from COPD except for ours until this report.

Moreover, in our 18-year longitudinal prospective study, we not only observed the decline of lung function in COPD and healthy controls who were COPD risk individuals, but we also identified the COPD susceptibility loci and their relationship with lung function. Distinguishable from other GWA studies on cross-sectional cohorts, our study was performed on a longitudinal prospective cohort and was replicated in rural and urban cohorts. The genetic variants were identified as being associated with an increased risk of COPD in the subjects between cases and controls in 1992. After 18 years, some of the COPD risk healthy controls in 1992 were diagnosed COPD after the lung function measurements in 2010 accompanied with those still in health without any interventions of cigarette smoking. The genetic variants which are associated with an increased COPD risk are considered to play important roles in the development of COPD. The identification of variants associated with increased COPD risk was also applied in populations of newly developed COPD patients and controls similar to the cases and controls in 2010. The common genetic variants of these identifications are possibly more credible for bias on the cross-sectional studies and would reduce these to a minimum. This was the first study to perform a genome-wide association examination on diseases that relate to genetic predispositions in a prospective study that was focused on the same population with 18 years of follow-up observations.

In the replication stage, there were two replication cohorts in which individuals were from rural and urban areas in Hubei, China respectively. These were carried on individuals from different environmental and living conditions to replicate the association of the polymorphisms of SNPs which were identified in the prospective stage with the risk of COPD in different circumstances and areas. COPD is a chronic pulmonary disease that involved the chronic inflammation in airways, which is a complex disease strongly affected by circumstance for the lung, including smoking addition, and genetic predisposition. Besides cigarette smoking, air conditions and certain living lifestyles may also influence the prevalence of COPD. As we have already adjusted for smoking history in our study, it was necessary to confirm the association of genetic variants in the regions with COPD in different circumstances for living in the replication stage. Thus, the replication stage in our study was performed in rural and urban areas to be more authentic and reliable.

However, the limitation in this study is that changes in smoking habits over time may effect lung function, and the benefits of smoking cessation in attenuating pulmonary function decline have been previously studied extensively [32]. Unfortunately, our study at present was not adequate to further confirm whether altering smoking habits is associated with changes in lung function. Since we recruited participants with smoking history of more than 20 pack-years and performed non-intervention in cigarette smoking during the follow-ups, all the subjects in each group of our study had nearly analogous smoking history. In addition, our study covered extensive follow-up data including a total of 2872 subjects, three different areas in china, 18- or 2-year follow-up observations. Despite this limitation, our study was adequately powered to identify the association between the two SNPs (rs7654947 and rs7671167) and lung function decline.

## Conclusions

Our genome-wide survey identified 2 new SNPs (rs11100865 and rs7654947) in HHIP that were associated with COPD susceptibility. Moreover, we found rs7654947 in HHIP and rs7671167 in FAM13A were related to decline in lung function. For the first time, we used a longitudinal prospective study with 18-year follow-up observations, to identify new loci both associated with COPD susceptibility and lung function decline in a Chinese Han population. Further GWAS will be required to investigate more candidate SNPs for COPD risk prediction.

## Additional file

Additional file 1: Methods: Specific details of meta-analyses in SNPs selected part.

## Abbreviations

COPD: Chronic obstructive pulmonary disease
HHIP: Human Hedgehog interacting protein
FAM13A: Family with sequence similarity 13 member A gene
GWAS: Genome-wide association studies
FEV_1_: Forced expiratory volume in one second
LD: linkage disequilibrium
SNPs: Single nucleotide polymorphisms
OR: Odds ratio
CI: 95% Confidence interval
