# Supplementary material for: Gene susceptibility identification in a longitudinal study confirms new loci in the development of chronic obstructive pulmonary disease and influences lung function decline
Source: Respir Res. 2015 Apr 18;16(1):49. doi: 10.1186/s12931-015-0209-3 (PMC4427922; doi:10.1186/s12931-015-0209-3)
Supplement: Additional file 1: — Methods: Specific details of meta-analyses in SNPs selected part. [file 12931_2015_209_MOESM1_ESM.pdf]

Additional file 1

Supplementary Methods

Meta-analyses

Totaling 16 SNPs were genotyped in our study. Five candidate SNPs among them were selected by meta-analyses, which were supposed to be associated with lung function or COPD susceptibility. Specifically, rs1828591 [1-3], rs12504628 [3, 4] and rs13118928 [1, 3, 5, 6] in HHIP, rs7671167 [5-8] in FAM13A, and rs13180 [5-7, 9] in IREB2 were included in the meta-analyses. Particular details can be accessed via the following supplementary figure S1 and references.

Figure S1. Specific details of meta-analyses.

rs1828591

Study or Subgroup	log[Odds Ratio]	SE	Weight	Odds Ratio IV, Fixed, 95% CI
Bergen cohort[1]	-0.3205	0.0848	22.7%	0.73 [0.61, 0.86]
Kim 2013[2]	-0.1313	0.1727	5.5%	0.88 [0.63, 1.23]
Rotterdam study[1]	-0.1715	0.0577	49.1%	0.84 [0.75, 0.94]
Wang 2013[3]	-0.0789	0.0847	22.8%	0.92 [0.78, 1.09]
**Total (95% CI)**			**100.0%**	**0.83 [0.77, 0.90]**

Heterogeneity: Chi² = 4.27, df = 3 (P = 0.23); I² = 30%
Test for overall effect: Z = 4.51 (P < 0.00001)

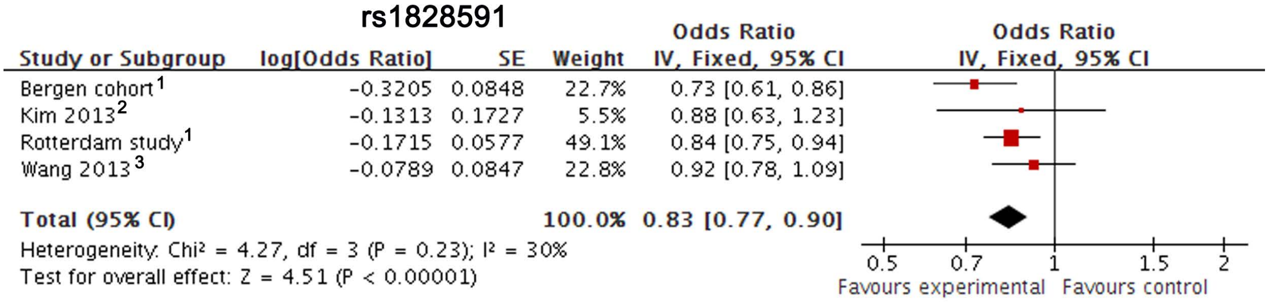

rs12504628

Study or Subgroup	log[Odds Ratio]	SE	Weight	Odds Ratio IV, Fixed, 95% CI
Soler Artigas 2011[4]	0.174	0.0309	88.1%	1.19 [1.12, 1.26]
Wang 2013[3]	0.0883	0.0842	11.9%	1.09 [0.93, 1.29]
**Total (95% CI)**			**100.0%**	**1.18 [1.11, 1.25]**

Heterogeneity: Chi² = 0.91, df = 1 (P = 0.34); I² = 0%
Test for overall effect: Z = 5.65 (P < 0.00001)

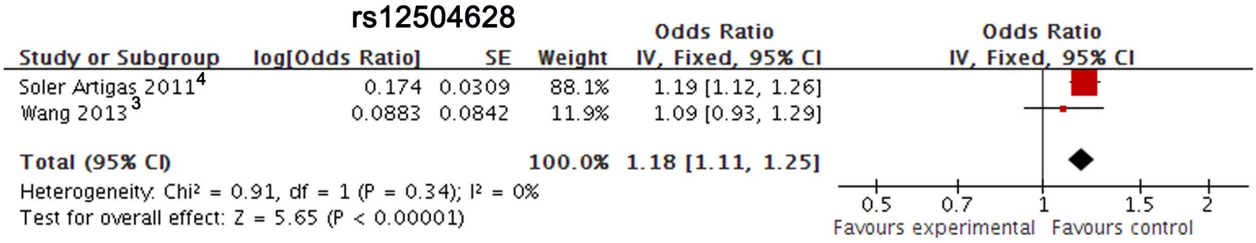

rs13118928

Study or Subgroup	log[Odds Ratio]	SE	Weight	Odds Ratio IV, Fixed, 95% CI
Bergen cohort[1]	-0.3205	0.0849	14.9%	0.73 [0.61, 0.86]
COPD-Gene[5]	-0.0834	0.0975	11.3%	0.92 [0.76, 1.11]
ECLIPSE[6]	-0.3011	0.1243	6.9%	0.74 [0.58, 0.94]
GenKOLS[5]	-0.2485	0.0852	14.8%	0.78 [0.66, 0.92]
NETT-NAS[5]	-0.3425	0.1211	7.3%	0.71 [0.56, 0.90]
Rotterdam study[1]	-0.2107	0.0601	29.7%	0.81 [0.72, 0.91]
Wang 2013[3]	-0.0818	0.0845	15.0%	0.92 [0.78, 1.09]
**Total (95% CI)**			**100.0%**	**0.81 [0.76, 0.86]**

Heterogeneity: Chi² = 7.60, df = 6 (P = 0.27); I² = 21%
Test for overall effect: Z = 6.56 (P < 0.00001)

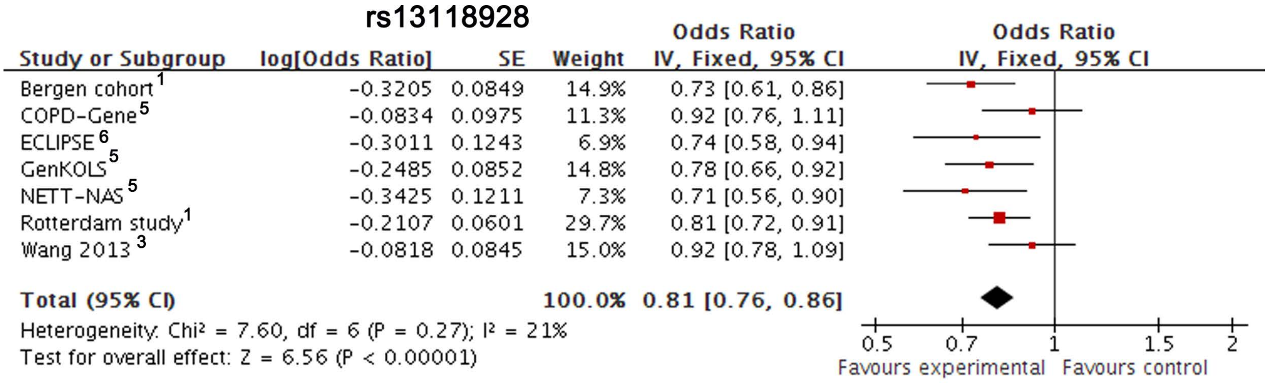

rs7671167

Study or Subgroup	log[Odds Ratio]	SE	Weight	Odds Ratio IV, Fixed, 95% CI
COPD-Gene[5]	-0.2485	0.1009	16.3%	0.78 [0.64, 0.95]
ECLIPSE[6]	-0.3285	0.1192	11.7%	0.72 [0.57, 0.91]
GenKOLS[5]	-0.2744	0.0877	21.6%	0.76 [0.64, 0.90]
Megan 2013[7]	-0.1985	0.1185	11.8%	0.82 [0.65, 1.03]
NETT-NAS[5]	-0.462	0.1282	10.1%	0.63 [0.49, 0.81]
Wang 2013[8]	-0.1061	0.0766	28.4%	0.90 [0.77, 1.05]
**Total (95% CI)**			**100.0%**	**0.79 [0.73, 0.85]**

Heterogeneity: Chi² = 6.89, df = 5 (P = 0.23); I² = 27%
Test for overall effect: Z = 5.85 (P < 0.00001)

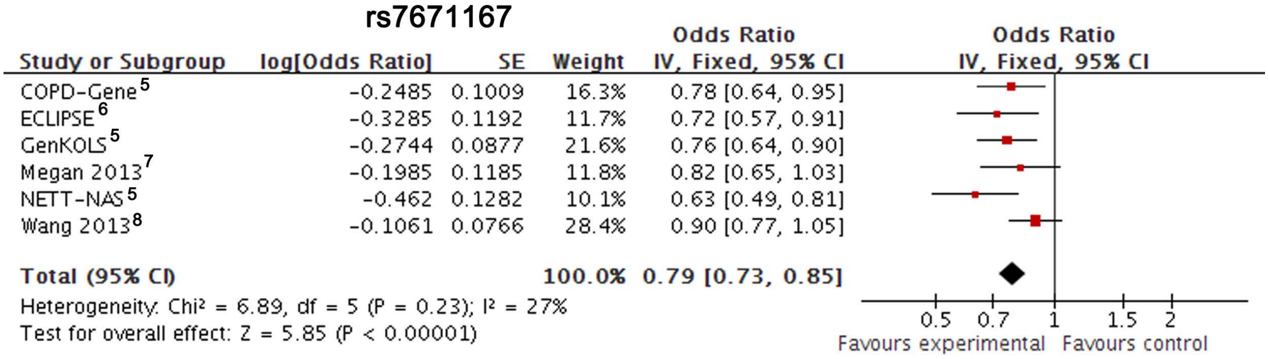

rs13180

Study or Subgroup	log[Odds Ratio]	SE	Weight	Odds Ratio IV, Fixed, 95% CI
COPD-Gene[5]	-0.2485	0.1009	17.4%	0.78 [0.64, 0.95]
ECLIPSE[6]	-0.2485	0.1254	11.3%	0.78 [0.61, 1.00]
GenKOLS[5]	-0.2357	0.0841	25.0%	0.79 [0.67, 0.93]
Megan 2013[7]	-0.3711	0.1346	9.8%	0.69 [0.53, 0.90]
NETT-NAS[5]	-0.3011	0.1243	11.5%	0.74 [0.58, 0.94]
Zhou 2012[9]	-0.0979	0.084	25.1%	0.91 [0.77, 1.07]
**Total (95% CI)**			**100.0%**	**0.80 [0.73, 0.87]**

Heterogeneity: Chi² = 3.95, df = 5 (P = 0.56); I² = 0%
Test for overall effect: Z = 5.36 (P < 0.00001)

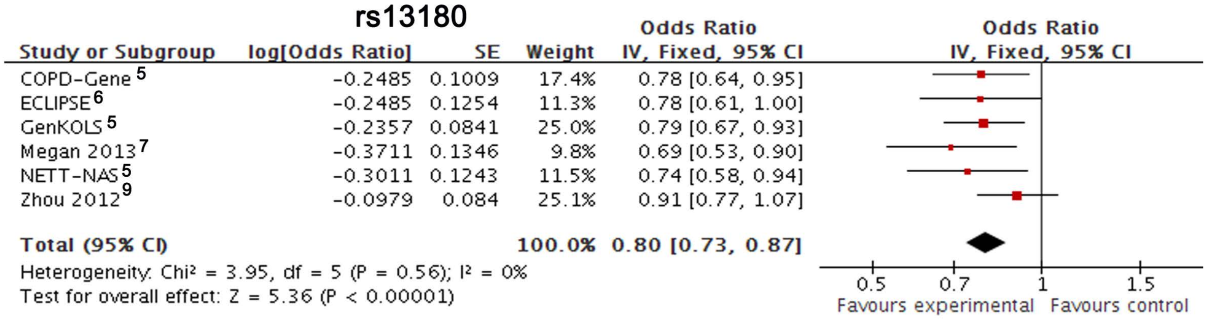

Supplementary Figure Legends

Figure S1. Totaling 3 SNPs (rs1828591, rs12504628, rs13118928) in HHIP, 1 SNP (rs7671167) in FAM13A and 1 SNP (rs13180) in IREB2 were supposed to be associated with lung function or COPD susceptibility in meta-analyses.

Supplementary References

1. Van Durme YM, Eijgelsheim M, Joos GF, Hofman A, Uitterlinden AG, Brusselle GG, Stricker BH: **Hedgehog-interacting protein is a COPD susceptibility gene: the Rotterdam Study.** *Eur Respir J* 2010, **36:**89-95.

2. Kim WJ, Oh YM, Lee JH, Park CS, Park SW, Park JS, Lee SD: **Genetic variants in HHIP are associated with FEV1 in subjects with chronic obstructive pulmonary disease.** *Respirology* 2013, **18:**1202-1209.

3. Wang B, Zhou H, Yang J, Xiao J, Liang B, Li D, Zhou H, Zeng Q, Fang C, Rao Z, et al: **Association of HHIP polymorphisms with COPD and COPD-related phenotypes in a Chinese Han population.** *Gene* 2013, **531:**101-105.

4. Soler Artigas M, Wain LV, Repapi E, Obeidat M, Sayers I, Burton PR, Johnson T, Zhao JH, Albrecht E, Dominiczak AF, et al: **Effect of five genetic variants associated with lung function on the risk of chronic obstructive lung disease, and their joint effects on lung function.** *Am J Respir Crit Care Med* 2011, **184:**786-795.

5. Cho MH, Castaldi PJ, Wan ES, Siedlinski M, Hersh CP, Demeo DL, Himes BE, Sylvia JS, Klanderman BJ, Ziniti JP, et al: **A genome-wide association study of COPD identifies a susceptibility locus on chromosome 19q13.** *Hum Mol Genet* 2012, **21:**947-957.

6. Pillai SG, Kong X, Edwards LD, Cho MH, Anderson WH, Coxson HO, Lomas DA, Silverman EK, Eclipse, Investigators I: **Loci identified by genome-wide association studies influence different disease-related phenotypes in chronic obstructive pulmonary disease.** *Am J Respir Crit Care Med* 2010, **182:**1498-1505.

7. Hardin M, Zielinski J, Wan ES, Hersh CP, Castaldi PJ, Schwinder E, Hawrylkiewicz I, Sliwinski P, Cho MH, Silverman EK: **CHRNA3/5, IREB2, and ADCY2 are associated with severe chronic obstructive pulmonary disease in Poland.** *Am J Respir Cell Mol Biol* 2012, **47:**203-208.

8. Wang B, Liang B, Yang J, Xiao J, Ma C, Xu S, Lei J, Xu X, Liao Z, Liu H, et al: **Association of FAM13A polymorphisms with COPD and COPD-related phenotypes in Han Chinese.** *Clin Biochem* 2013, **46:**1683-1688.

9. Zhou H, Yang J, Li D, Xiao J, Wang B, Wang L, Ma C, Xu S, Ou X, Feng Y: **Association of IREB2 and CHRNA3/5 polymorphisms with COPD and COPD-related phenotypes in a Chinese Han population.** *J Hum Genet* 2012, **57:**738-746.
